# Plasma gelsolin levels are associated with diabetes, sex, race, and poverty

**DOI:** 10.1186/s12967-023-04026-5

**Published:** 2023-03-10

**Authors:** Nicole Noren Hooten, Nicolle A. Mode, Edward Kowalik, Victor Omoniyi, Alan B. Zonderman, Ngozi Ezike, Mark J. DiNubile, Susan L. Levinson, Michele K. Evans

**Affiliations:** 1 Laboratory of Epidemiology and Population Sciences, National Institute on Aging, National Institutes of Health, NIH Biomedical Research Center, 251 Bayview Boulevard, Suite 100, Baltimore, MD 21224 USA; 2 BioAegis Therapeutics, North Brunswick, NJ USA; 3 Present Address: Johns Hopkins School of Medicine, Baltimore, MD USA

**Keywords:** Diabetes mellitus, pGSN, Extracellular vesicles (EV), African American, Poverty, Race, Sex, Social determinants of health, Poverty, Inflammation

## Abstract

**Background:**

The growing epidemic of the inflammation-related metabolic disease, type 2 diabetes mellitus, presents a challenge to improve our understanding of potential mechanisms or biomarkers to prevent or better control this age-associated disease. A gelsolin isoform is secreted into the plasma as part of the extracellular actin scavenger system which serves a protective role by digesting and removing actin filaments released from damaged cells. Recent data indicate a role for decreased plasma gelsolin (pGSN) levels as a biomarker of inflammatory conditions. Extracellular vesicles (EVs), a heterogeneous group of cell-derived membranous structures involved in intercellular signaling, have been implicated in metabolic and inflammatory diseases including type 2 diabetes mellitus. We examined whether pGSN levels were associated with EV concentration and inflammatory plasma proteins in individuals with or without diabetes.

**Methods:**

We quantified pGSN longitudinally (n = 104) in a socioeconomically diverse cohort of middle-aged African American and White study participants with and without diabetes mellitus. Plasma gelsolin levels were assayed by ELISA. EV concentration (sub-cohort n = 40) was measured using nanoparticle tracking analysis. Inflammatory plasma proteins were assayed on the SomaScan® v4 proteomic platform.

**Results:**

pGSN levels were lower in men than women. White individuals with diabetes had significantly lower levels of pGSN compared to White individuals without diabetes and to African American individuals either with or without diabetes. For adults living below poverty, those with diabetes had lower pGSN levels than those without diabetes. Adults living above poverty had similar pGSN levels regardless of diabetes status. No correlation between EV concentrations and pGSN levels was identified (r = − 0.03; p = 0.85). Large-scale exploratory plasma protein proteomics revealed 47 proteins that significantly differed by diabetes status, 19 of which significantly correlated with pGSN levels, including adiponectin.

**Conclusions:**

In this cohort of racially diverse individuals with and without diabetes, we found differences in pGSN levels with diabetes status, sex, race, and poverty. We also report significant associations of pGSN with the adipokine, adiponectin, and other inflammation- and diabetes-related proteins. These data provide mechanistic insights into the relationship of pGSN and diabetes.

## Background

The growing epidemic of type 2 diabetes is rapidly becoming a world-wide problem. In 2019 it was estimated that 463 million adults were living with diabetes mellitus [1]. These numbers are forecasted to rise by 51% in the next 26 years [1], indicating that diabetes mellitus is projected to continue to be a global public health problem. Therefore, it is important to fully understand the underlying mechanisms that drive diabetes and pursue novel therapeutic modalities to combat this prevalent disease. Diabetes is characterized by impaired insulin secretion coupled with insulin resistance. The pathophysiology of insulin resistance involves inflammatory mechanisms, mitochondrial dysfunction, and hormonal dysregulation (e.g., adipokines) [2]. Thus, examining plasma proteins that may play a role in inflammatory processes may advance our understanding of the risk profiles for this metabolic age-associated disease.

Gelsolin exists as both a cytoplasmic and extracellular form, but only the extracellular form contains a “plasma extension” 24 amino acid sequence and a disulfide bond that augments stability of this isoform in the extracellular environment [3, 4]. This extracellular isoform, called plasma gelsolin (pGSN), is part of the extracellular actin scavenger system (EASS) and functions to continuously scavenge and remove actin filaments in the extracellular space, including the circulation. Cellular injury can release cellular debris, including actin filaments and DNA, into the extracellular space. To avoid the toxicity of insoluble F-actin filaments, the increase in blood viscosity, platelet activation, and induction of proinflammatory molecules, compensatory mechanisms are in place to scavenge and clear extracellular actin as part of the EASS. pGSN functions to depolymerize actin by severing and capping actin filaments, thereby facilitating its removal from the circulation [3, 4]. Actin inhibits DNase and gelsolin disinhibits DNase; thus, gelsolin may also promote the clearance of cell-free DNA [5].

Recent data suggest that pGSN may have a vital role in both normal physiological and pathological processes. In general, lower levels of pGSN have been reported in response to trauma, sepsis, chronic inflammatory diseases including rheumatoid arthritis, specific neurological disorders including Alzheimer’s disease and multiple sclerosis, and some cancers [4, 6]. Substantial interest has arisen in utilizing decreased pGSN as a prognostic biomarker [7, 8]. Given the beneficial role of pGSN repletion in diverse animal models, a recombinant human pGSN is actively being developed for therapeutic use in a diverse variety of infectious and other inflammatory conditions.

Small exploratory studies have reported differences in pGSN with diabetes. A study using liquid chromatography-mass spectrometry (LC–MS)–based proteomics to identify plasma proteins reported lower levels of pGSN in adolescent/young adults (average age 19.4 yrs) with type 1 diabetes [9]. This study also found lower levels of pGSN in adolescents (average age 14.4 yrs) with type 2 diabetes compared to healthy controls (average age 20.7 yrs) [9]. In a small cohort of obese women with (n = 6) or without (n = 6) diabetes, pGSN levels were lower after bariatric surgery [10]. Another recent study reported lower levels of pGSN in women with type 2 diabetes (n = 10) compared to women without type 2 diabetes (n = 8) [11]. Similar results were reported for men with type 2 diabetes (n = 17) compared to men without type 2 diabetes (n = 7). In this study, it was also found that two different mouse models of diabetes, *db/db* (homozygous for the diabetes spontaneous mutation (*Lepr*^*db*^)) mice and mice on a High Fat Diet (HFD) + Streptozotocin, also reported reduced pGSN compared to control mice [11]. Therefore, these studies together suggest that there may be differences in pGSN levels with and without diabetes, but more studies are warranted to confirm these findings. For example, few studies have measured pGSN levels over time and in a large, diverse cohort of middle-aged adults.

Extracellular vesicles (EVs) represent another tool to examine underlying pathophysiology of diabetes mellitus [12]. EVs are a diverse group of cell-derived membranous structures that include exosomes, microvesicles, and apoptotic bodies that are released by cells [13, 14]. In decompression illness, microparticles increase with an associated drop in pGSN levels [15]. EVs may be found in numerous bodily fluids including plasma, serum, and urine [13, 14]. They contain molecular cargo, including several types of RNAs, nuclear DNA, mitochondrial DNA, proteins, and lipids [14–19]. EVs mediate intercellular communication and are also associated with multiple immune, metabolic, and neoplastic diseases including diabetes mellitus [12–14, 20]. Our previous work found higher levels of circulating EVs in White adults with diabetes compared to those without diabetes or to African Americans with or without diabetes, suggesting that EVs may play a role in diabetes mellitus differentially in certain demographic groups [21].

In this study, we examined the association of pGSN with diabetes mellitus in a large, diverse cohort of African American and White adults with or without diabetes mellitus. We also investigated whether there was a relationship between EV concentration and pGSN in diabetic individuals. Additionally, we examined how the social determinants of health, race and poverty, affect pGSN levels. Levels of pGSN were measured at two different time points to determine whether pGSN levels changed over time in this cohort.

## Methods

### Study sample

Participants were chosen from the Healthy Neighborhoods of Diversity across the Life Span (HANDLS) study of the National Institute on Aging Intramural Research Program, National Institutes of Health. HANDLS is an ongoing, prospective study that examines how race and socioeconomic status influence aging and age-related disease [22]. The study consists of African American and White adults aged 30–64 at baseline living in Baltimore, Maryland, USA. The baseline accrual occurred between 2004 and 2009. Race was self-identified as African American or White. Participants were either above or below poverty as defined by 125% of the 2004 U.S. Health and Human Services Poverty Guidelines at enrollment. Participants provided written informed consent. The HANDLS study is approved by the Institutional Review Board of the National Institutes of Health.

Each HANDLS visit occurs approximately every 5 years and consists of physical exams, structured medical history interviews, questionnaires, and collection of blood samples. Body mass index (BMI) was calculated as weight (kg) divided by height squared (m^2^). Individuals were classified based on BMI as underweight/normal (< 25 kg/m^2^), overweight/obese class I (25 to < 35 kg/m^2^), and obese class II/III (≥ 35 kg/m^2^). Individuals with diabetes met one of the following three criteria: (1) self-reported previous diagnosis by health care provider, (2) currently taking medication for diabetes, or (3) fasting serum glucose ≥ 126 mg/dL. For the study sample, we chose participants who had fasting blood samples at two times ~ 5 years apart. These time points corresponded to wave 3 and wave 4 of the HANDLS study, which we will refer to here as time 1 and time 2. We randomly selected 26 African American and 26 White participants with diabetes at time 1, and randomly selected 26 African American and 26 White euglycemic participants at time 1 who were matched on BMI category and 10-yr age groups. Participants were aged 40 and older at time 1 and were excluded if diagnosed with HIV. Therefore, the final cohort consisted of 104 participants: 52 euglycemic adults and 52 individuals with diabetes with longitudinal samples at two time points (Table 1). Among euglycemic participants living below poverty, 47% were African American; among those living above poverty, 54% were African American. Among participants with diabetes living below poverty, 47% were African American; among those living above poverty 51% were African American.

**Table 1:** HANDLS longitudinal subcohort for plasma gelsolin measurements

Subcohort	Euglycemic (N = 52)	Diabetes (N = 52)	p-value^a^
Men, N (%)	18 (35)	24 (46)	0.318
African American, N (%)	26 (50)	26 (50)	1.000
Below Poverty, N (%)	22 (42)	29 (56)	0.239
Age at Time 1, mean (SD)	55.5 (7.5)	55.9 (6.4)	0.778
pGSN at Time 1, mean (SD)	44.0 (7.9)	40.2 (7.7)	0.015
BMI, mean (SD)	30.7 (6.7)	32.9 (9.0)	0.158

Data by time point	Time 1	Time 2	p-value^a^
Age, mean (SD)	55.7 (7.0)	59.6 (7.3)	< 0.001
BMI, mean (SD)	31.8 (7.9)	31.9 (8.4)	0.943
pGSN, mean (SD)	42.1 (8.0)	42.8 (9.1)	0.565

^a^Test of differences for categorial variables used the Pearson Chi-squared test. Test of differences for continuous variables used a Student’s t-test

pGSN: plasma gelsolin; SD: standard deviation

### Gelsolin measurement

Fasting blood samples were collected between 9:30 and 11:30 a.m. into sodium heparin blood collection tubes. Histopaque®-1077 (2.5 ml) was added to the bottom of the tube containing 5 ml blood and centrifuged for 20 min at 610*g* at 4 °C without the brake. Tubes were visually inspected for proper separation and hemolysis. The top layer containing plasma was aliquoted and stored at − 80 °C.

Gelsolin was measured by BioAegis® Therapeutics by a proprietary ELISA using a primary antibody raised against the N terminal tail specific to plasma gelsolin [8]. Samples from the different visits were run on the same plate for each individual, with the exception of nine samples. Plates were balanced to contain individuals across race and diabetes diagnosis across all plates. Each sample was run in triplicate, and the sample average was used. The coefficient of variance (CV) for sample replicates was 3.73% (time 1) and 3.49% (time 2). Controls (QC) were produced by spiking recombinant human plasma gelsolin into a plasma sample at three different concentrations 10 ng/ml, 50 ng/ml and 100 ng/ml; the same donor plasma was used for the entire study. In addition, the 50 ng/ml spike-in control (QC-M) was aliquoted, stored at − 80 °C and run on each subsequent plate. The mean intra-assay CV was 3.64% and 3.99% for QC and QC-M respectively. The mean inter-assay CV was 6.05% and 6.19%, respectively.

### Plasma EV isolation

Of the initial pGSN cohort, we chose a subset of 20 White participants with diabetes and 20 White participants without diabetes to examine plasma EV concentration. Plasma (0.5 ml) from fasting participants was thawed on ice and EVs were isolated using ExoQuick^TM^ Exosome Precipitation Solution (System Bioscience Inc.). Details about EV isolation have been described previously [23]. The EV pellet was resuspended in PBS and diluted 1:300 in PBS for NTA. All samples were then stored at − 80 °C until use.

### Nanoparticle tracking analysis

Diluted EVs were thawed, and concentration was measured using nanoparticle tracking analysis on a NanoSight NS500 (Malvern Instruments Ltd.) according to manufacturer’s instructions by a single operator, blinded to sample identity. For each EV sample, five 20 s videos were recorded at camera level = 15. Analysis was performed using NTA 3.4 Build 3.4.4 software at detection level = 4. Calculations for plasma EV concentration were described previously [23].

### SomaScan®

Of the initial pGSN cohort, we chose a subset of White participants (n = 18) with diabetes and White participants without diabetes (n = 18) to utilize plasma samples for SomaScan assays. These individuals were also in our EV subcohort. Plasma samples were run on the SomaScan® v4 proteomic platform by SomaLogic, Inc (Boulder, CO). Quality control, sample calibration and normalization were performed by SomaLogic. Only markers for human proteins were considered. We then estimated the limit of detection (LoD) using SomaScan Assay qualification that variance for buffer samples is approximately the same as variance for low protein quantity samples [24]. Markers with ≥ 30% of samples below the LoD were removed (3%). Relative fluorescence units (RFU) for each protein were then log_10_ transformed and centered and scaled into z-scores. A total of 7069 markers were available for analysis.

### Statistics

Statistical analyses were performed using R, version 4.2.0 [25]. Differences between groups were tested using Student's t-test for continuous variables and chi-squared tests of independence for categorical variables. Linear mixed model regression (package lme4) examined the associations of diabetes status with longitudinal measurements of gelsolin accounting for race, sex, poverty status, BMI category and age. Backward elimination was used for model building starting with the full model of interactions among diabetes status with race, sex, age, and poverty status. Significance of fixed factors were determined by log likelihood ratio tests. Statistical significance was defined as p-value < 0.05.

SomaScan proteins which met the data criteria (see SomaScan section) were analyzed using Student’s t-tests to compare mean RFU values between participants with diabetes and euglycemic participants. Effect size was determined using Cohen’s D. Significant markers were those which resulted in a p-value < 0.01 and an effect size ≥ 1. Pearson’s correlation (r) was used to examine the relationship between protein markers and pGSN.

## Results

To examine the relationship between pGSN levels and diabetes, we designed a longitudinal cohort of African American and White individuals with or without diabetes (Table 1). Overall, 104 HANDLS participants with or without diabetes were included in this study, matched on race, sex, BMI category and 10-yr age groups (Table 1). Per the matched design, there were no differences between groups in the distributions of age and BMI (Table 1). There were also no differences in sex, race, and poverty status. Euglycemic participants had significantly higher pGSN levels than diabetic participants. Overall, pGSN levels were 42.08 ± 7.99 µg/ml and 42.77 ± 9.12 µg/ml at time 1 and 2, respectively.

**Fig. 1:**
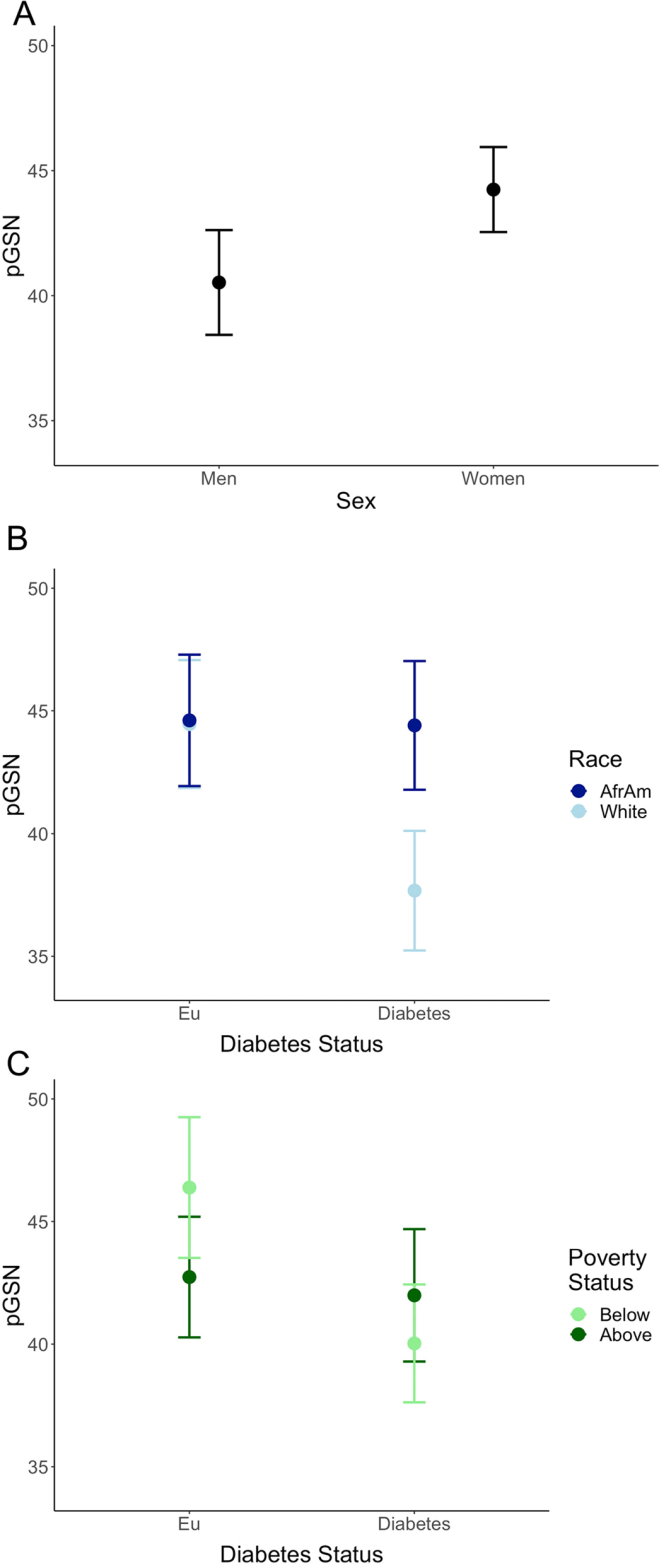
Association of plasma gelsolin, diabetes status and demographic variables. Plasma gelsolin (pGSN) levels were measured in a cohort of 104 euglycemic (Eu) individuals and 104 individuals with diabetes at 2 different time points ~ 5 years apart. Linear mixed model regression accounting for repeated measurements was used to analyze the relationship of pGSN, diabetes status, sex (**A**), race (**B**), and poverty status (**C**). AfrAm: African American

Using linear mixed model regression, we found that pGSN levels were significantly lower in men than women (p = 0.007, Fig. 1A). We also found a significant race and diabetes interaction (p = 0.012; Fig. 1B). White individuals with diabetes had significantly lower levels of pGSN compared to White individuals without diabetes or compared to African American individuals either with or without diabetes. pGSN levels were similar for African American participants with and without diabetes. There was also a significant interaction between poverty status and diabetes status (p = 0.033; Fig. 1C). For adults living below poverty, those with diabetes had lower pGSN levels than those without diabetes. Adults living above poverty had similar pGSN levels regardless of diabetes status. There were no significant interactions with age, indicating that the rates of change were similar regardless of demographics or diabetes status.

To gain a better understanding of the mechanisms that may account for differences in pGSN levels in individuals with diabetes, we first examined whether pGSN levels were related to circulating levels of extracellular vesicles (EVs). Previously in a different cohort, we found that plasma EVs were higher in White individuals with diabetes compared to White individuals without diabetes or compared to African American individuals either with or without diabetes [21]. Here, we speculated that EVs may function as physical “sponges” resulting in reduced levels of pGSN. From our pGSN cohort, we chose to isolate EVs from White individuals as this group displayed differences in pGSN levels with diabetes status. Plasma EVs were isolated from 20 White individuals with diabetes and 20 euglycemic White individuals. We found that there was no correlation between EV concentration and pGSN (r = − 0.03; p = 0.85). In light of significant differences in each of these variables by diabetes status, the lack of correlation between EV and pGSN levels may be due to the different tests employed. Correlation assesses the preservation of rank order between continuous variables, while tests between diabetes groups assess overall mean differences. Therefore, circulating EVs levels are not related to pGSN levels in this subcohort.

We further investigated the relationship of pGSN to other circulating proteins by performing an exploratory, unbiased proteomic approach to examine > 7000 circulating proteins using SomaScan®, which is a sensitive, reproducible assay with high specificity for detecting proteins in a large dynamic range. Data processing details are listed in Materials and Methods. We found 47 proteins that were significantly different between euglycemic individuals and individuals with diabetes (Table 2). Of these 47, we then examined if any of these proteins were correlated with pGSN levels. We found that there were 19 proteins that were significantly correlated with pGSN (indicated in bold in Table 2). Except for insulin-like peptide INSL6, these proteins were positively correlated with pGSN levels (Table 2). One of these proteins, adiponectin, is an adipokine that is secreted from adipocytes and plays a key role in metabolic processes and in obesity-related diseases, including diabetes [26].

**Table 2:** Plasma proteins that differ significantly by diabetes status and their correlation with plasma gelsolin

Protein name	Entrez gene symbol	Diabetes t-test p-value	Effect size	Correlation (r) with pGSN	Correlation p-value
Anthrax toxin receptor 2	ANTXR2	0.0014	1.0142	0.6038	**0.0001**
Matrix-remodeling-associated protein 8:Extracellular domain	MXRA8	0.0005	1.0924	0.5692	**0.0003**
Alpha-1,6-mannosylglycoprotein 6-beta-N-acetylglucosaminyltransferase A	MGAT5^1^	0.0007	1.0697	0.5655	**0.0003**
Cartilage oligomeric matrix protein	COMP	0.0007	1.0689	0.5595	**0.0004**
Alpha-1,6-mannosylglycoprotein 6-beta-N-acetylglucosaminyltransferase A	MGAT5^2^	0.0005	1.0888	0.5144	**0.0013**
Histone-lysine N-methyltransferase EHMT2	EHMT2	0.0013	1.0183	0.4649	**0.0043**
Transmembrane protein 132C:Extracellular domain	TMEM132C	0.0003	1.1285	0.4560	**0.0052**
Adiponectin	ADIPOQ	0.0001	1.2230	0.4513	**0.0057**
Brevican core protein	BCAN	0.0007	1.0629	0.4334	**0.0083**
Vesicular, overexpressed in cancer, prosurvival protein 1	VOPP1	0.0003	1.1278	0.4299	**0.0089**
Iduronate 2-sulfatase	IDS	0.0015	1.0072	0.4183	**0.0111**
72 kDa type IV collagenase	MMP2	0.0008	1.0538	0.3861	**0.0200**
Peptidyl-glycine alpha-amidating monooxygenase	PAM	0.0007	1.0729	0.3625	**0.0298**
Dihydrolipoyl dehydrogenase, mitochondrial	DLD^3^	0.0000	1.2480	0.3602	**0.0309**
1-phosphatidylinositol-4,5-bisphosphate phosphodiesterase delta-1	PLCD1	0.0017	1.0016	0.3599	**0.0311**
Prosaposin	PSAP	0.0013	1.0460	0.3595	**0.0313**
Neurogenic locus notch homolog protein 3	NOTCH3	0.0008	1.0683	0.3463	**0.0386**
Acetylcholinesterase	ACHE	0.0009	1.0424	0.3347	**0.0460**
Complement C1q tumor necrosis factor-related protein 5	C1QTNF5	0.0015	1.0095	0.3290	0.0501
Calsyntenin-1	CLSTN1	0.0011	1.0411	0.3266	0.0518
Glypican-3	GPC3	0.0016	1.0006	0.3229	0.0548
Protein Wnt-5b	WNT5B	0.0003	1.1235	0.3126	0.0634
Glutathione reductase, mitochondrial	GSR	0.0016	1.0056	0.3006	0.0748
Dihydrolipoyl dehydrogenase, mitochondrial	DLD^4^	0.0002	1.1495	0.2651	0.1182
Arylsulfatase A	ARSA	0.0007	1.0896	0.2050	0.2304
Dermokine	DMKN	0.0007	1.0638	0.1446	0.4002
Glutathione synthetase	GSS	0.0008	1.0542	0.0459	0.7905
Cyclic AMP-responsive element-binding protein 3-like protein 4	CREB3L4	0.0007	1.0592	− 0.0158	0.9271
Protein phosphatase 1 regulatory subunit 14A	PPP1R14A	0.0006	1.0902	− 0.0782	0.6505
Sulfatase-modifying factor 1	SUMF1	0.0004	1.1168	− 0.0941	0.5853
Ribonuclease 4	RNASE4	0.0001	1.1939	− 0.0970	0.5735
SH2 domain-containing protein 1A	SH2D1A	0.0007	1.0685	− 0.1072	0.5336
Cyclin-dependent kinase 15; EC = 2.7.11.22	CDK15	0.0009	1.0487	− 0.1344	0.4345
Apoptosis-associated speck-like protein containing a CARD	PYCARD	0.0007	1.0588	− 0.1366	0.4270
Protocadherin gamma-C3	PCDHGC3	0.0011	1.0315	− 0.1539	0.3702
Trafficking protein particle complex subunit 3	TRAPPC3	0.0013	1.0145	− 0.1561	0.3631
Ephrin-A1	EFNA1	0.0010	1.0422	− 0.1580	0.3575
Neural proliferation differentiation and control protein 1	NPDC1	0.0007	1.0657	− 0.1769	0.3019
Delta-like protein 1	DLL1	0.0001	1.1820	− 0.1792	0.2956
Guanine nucleotide exchange factor VAV3	VAV3	0.0004	1.1155	− 0.2064	0.2272
Protein FAM3B	FAM3B	0.0010	1.0383	− 0.2271	0.1828
Vascular non-inflammatory molecule 2	VNN2	0.0006	1.0792	− 0.2457	0.1485
Calsequestrin-1	CASQ1	0.0011	1.0292	− 0.2478	0.1450
Protein kinase C gamma type	PRKCG	0.0002	1.1413	− 0.2723	0.1082
Plasma serine protease inhibitor	SERPINA5	0.0014	1.0203	− 0.2972	0.0783
Rho GTPase-activating protein 36	ARHGAP36	0.0000	1.2910	− 0.3037	0.0718
Insulin-like peptide INSL6	INSL6	0.0001	1.1802	− 0.3769	**0.0234**

SomaScan was used to quantify plasma proteins and a Student’s t-test was used to examine differences between euglycemic and diabetic individuals. Cohen effect size is indicated. Pearson’s correlation (r) and p-value are indicated for the relationships between plasma proteins and pGSN. Proteins significantly correlated with pGSN are indicated in bold. Values are sorted by correlation with high positive values at top

Multiple markers can match to the same target protein and are uniquely identified by sequence numbers. 1. SomaScan seq. 21768.9; . 2. SomaScan seq. 21813.171; 3. SomaScan seq. 10025.1 ; 4. SomaScan seq. 15527.90

## Discussion

In this study, we examined pGSN levels in a large longitudinal cohort of African American and White adults with or without diabetes. We found that pGSN was associated with the social determinants of health, race and poverty status, in the context of diabetes. White adults with diabetes had lower pGSN levels compared to the White adults without diabetes, as well as compared to African American adults with or without diabetes. In addition, we report that for individuals living below poverty status, those with diabetes had lower levels of pGSN compared to euglycemic individuals. Given the beneficial role of pGSN in clearing toxic actin filaments and other inflammatory mediators from the circulation, these data suggest that White adults with diabetes and adults living below poverty with diabetes may be at higher risk for end organ complications given that these groups have the lowest level of pGSN.

The average pGSN levels in our cohort were 42.1 µg/ml at time 1 and 42.8 µg/ml at time 2. This level is lower than what has previously been reported for human cohorts and may be due to several reasons. First, the reported concentration of pGSN can vary widely depending on the methodology used for measurement [4]. Available commercial kits for measurement of gelsolin by ELISA have not reported validation data for clinical use, including matrix effects, and frequently do not standardize against purified protein. Western blot and other immunoblotting methods used to estimate pGSN tends to have higher values due to denaturing of the samples which removes matrix interference effects, but have high variability. Actin nucleation assays can also be used to indirectly measure pGSN, but these assays are technically more difficult to perform, and have not been successfully used in multiwell plates where quality control can be tested. Here, we used a sensitive, quantitative ELISA which is specific for the secreted, plasma form of gelsolin [8].

In our cohort, we found that pGSN levels were lower in men than in women. This is consistent with another study that reported lower pGSN in men compared to women in a cohort of ankylosing spondylitis patients undergoing anti-TNF-alpha therapy and controls [27]. However, very few reports have examined differences in pGSN with sex or race. Here we also report that White adults with diabetes have lower pGSN compared to White adults without diabetes as well as to African American adults either with or without diabetes. Our findings demonstrate that it is important to quantify pGSN levels in diverse cohorts to determine its predictive value as a biomarker of disease. In addition, we report differences in pGSN with poverty status. Individuals with diabetes living below poverty have the lowest level of pGSN. This finding is important since poverty and low socioeconomic status may enhance the risk for developing the well-known complications of diabetes mellitus, cardiovascular disease, chronic kidney disease and premature mortality [28–31]. Here we have identified pGSN as a potentially novel biological transducer of social disadvantage.

Previously, we reported higher levels of circulating EVs in White adults with diabetes, a finding not observed in African Americans with diabetes. As EVs can contain surface filamentous actin which can bind to pGSN [15], we postulated that EVs may bind and reduce circulating pGSN levels in White individuals with diabetes. Conversely, higher pGSN levels might reduce EV levels. However, contrary to our initial hypothesis, we did not find that pGSN levels were correlated with circulating EV levels in our cohort. This difference may be since EVs may contain surface filamentous actin only under specific injury or acute conditions. For example, EVs have been shown to bind to pGSN under stress conditions of high pressure and decompression [15]. In this study larger EVs were analyzed using flow cytometry. Therefore, there may be a subset of larger EVs that contain surface filamentous actin and bind to pGSN.

In our diverse cohort of euglycemic participants and participants with diabetes, we found that pGSN levels were significantly correlated with 19 plasma proteins. Of these proteins, several have previously been associated with diabetes. Most notably, adiponectin was positively correlated with pGSN. Both pGSN and adiponectin have roles in combating inflammation. Given the inflammatory component of diabetes, this data suggests that lower levels of pGSN and adiponectin in White adults with diabetes may lead to heightened inflammation in these individuals. Adiponectin and other proteins identified in our analysis, such as ARHGAP36, WNT5B, VAV3, Ephrin-A1 and MMP2 have roles in regulating and remodeling the vascular endothelium. Vascular dysfunction in individuals with type 2 diabetes contributes to the increased burden of cardiovascular disease and end organ complications [32, 33], which may suggest a mechanism whereby individuals with diabetes are at a higher risk for vascular comorbidities and end organ complications. In support of this idea, an exploratory study using iTRAQ proteomics followed by immunoblotting validation reported that pGSN were lower in individuals with type 2 incipient diabetic nephropathy compared to controls [34]. Since this study used immunoblotting as a method for semi-quantitative pGSN validation, this study is interesting but warrants further investigation and follow up.

One limitation to our study is that the SomaScan assays were performed in a smaller subset of the initial cohort. Therefore, the relationships that we discovered between pGSN and plasma proteins should be interpreted as exploratory. It is not known whether these relationships would persist in a larger cohort. Furthermore, the small sample size reduced statistical power to examine numerous covariates in the analysis. Nevertheless, as this is a large-scale proteomic profile examining pGSN with other plasma proteins, these data implicate a role of pGSN in diabetes. In addition, poverty status was ascertained at study baseline and may change over time. As is the case with many observational cohort studies, we cannot rule out residual confounding despite considering key variables when designing the cohort and also the inclusion of covariates into our analyses.

Our study has several notable strengths. First, we measured pGSN longitudinally at two different time points, which is important given that there are few studies with longitudinal measurements of pGSN. Second, our cohort consists of both African Americans and White adults living above and below poverty. In this study, race and poverty status were equally represented across diabetes groups. This enabled us to examine the effects of race and poverty status on pGSN in the context of diabetes.

## Conclusions

Here we report lower pGSN levels in White individuals with diabetes compared to White individuals without diabetes as well as compared to African American adults either with or without diabetes mellitus. In addition, pGSN levels were lower in individuals with diabetes living below poverty and in men compared to women. pGSN levels were significantly correlated with the adipokine, adiponectin and other inflammation- and diabetes-related proteins. These data may indicate a potential role of pGSN in diabetes and further our knowledge about the utility of pGSN as a biomarker of diabetes.

## Data Availability

The datasets generated and analyzed during the current study are available from the corresponding author on reasonable request through the HANDLS website https://handls.nih.gov/.

## Abbreviations

pGSN: Plasma gelsolin
BMI: Body mass index
EV: Extracellular vesicle
HANDLS: Healthy Aging in Neighborhoods of Diversity across the Life Span
LoD: Limit of detection
EASS: Extracelluar actin scavenger system
RFU: Relative fluorescence units
